# Assessment of KRAS^G12C^ inhibitors for colorectal cancer

**DOI:** 10.3389/fonc.2024.1412435

**Published:** 2024-06-24

**Authors:** Gary A. Piazza, Preethi Chandrasekaran, Yulia Y. Maxuitenko, Karim I. Budhwani

**Affiliations:** ^1^ Department of Drug Discovery and Development, Harrison College of Pharmacy, Auburn University, Auburn, AL, United States; ^2^ University of Texas (UT) Southwestern Medical Center, Dallas, TX, United States; ^3^ CerFlux, Birmingham, AL, United States; ^4^ University of Alabama at Birmingham, Birmingham, AL, United States

**Keywords:** colorectal cancer, KRAS G12C inhibitors, resistance, clinical trials, KRAS mutation G12C

## Abstract

Colorectal cancer (CRC) is a highly prevalent and lethal cancer worldwide. Approximately 45% of CRC patients harbor a gain-in-function mutation in KRAS. KRAS is the most frequently mutated oncogene accounting for approximately 25% of all human cancers. Gene mutations in KRAS cause constitutive activation of the KRAS protein and MAPK/AKT signaling, resulting in unregulated proliferation and survival of cancer cells and other aspects of malignant transformation, progression, and metastasis. While KRAS has long been considered undruggable, the FDA recently approved two direct acting KRAS inhibitors, Sotorasib and Adagrasib, that covalently bind and inactivate KRAS^G12C^. Both drugs showed efficacy for patients with non-small cell lung cancer (NSCLC) diagnosed with a KRAS^G12C^ mutation, but for reasons not well understood, were considerably less efficacious for CRC patients diagnosed with the same mutation. Thus, it is imperative to understand the basis for resistance to KRAS^G12C^ inhibitors, which will likely be the same limitations for other mutant specific KRAS inhibitors in development. This review provides an update on clinical trials involving CRC patients treated with KRAS^G12C^ inhibitors as a monotherapy or combined with other drugs. Mechanisms that contribute to resistance to KRAS^G12C^ inhibitors and the development of novel RAS inhibitors with potential to escape such mechanisms of resistance are also discussed.

## Introduction

Colorectal cancer (CRC) is the third most prevalent cancer and the second leading cause of cancer related mortality worldwide, according to Global Cancer Statistics 2018 (1). CRC is recognized as a heterogenous malignancy with a complex mutational landscape in which over 45% of cases harbor KRAS mutations but with additional mutations, for example, in components of the APC/β-catenin pathway. While only 3% of CRC patients are diagnosed with the KRAS^G12C^ mutation, this type of CRC is often associated with rapid progression and shorter overall survival rate compared to patients diagnosed with non-KRAS^G12C^ mutations (2–4). KRAS^G12C^ mutations result from a glycine-to-cysteine substitution at position 12 of KRAS protein leading to constitutive activation of KRAS (5).

Under physiological conditions, wild-type (WT) RAS functions enzymatically as a GTPase to regulate normal cell proliferation, for example, in the colonic mucosa to regenerate surface epithelium. RAS is often described as a molecular switch that is “off” when bound to GDP or “on” when GTP is bound, whereby “off” and “on” refer to different conformations of RAS that regulate its capacity to bind effectors such as RAF or PI3K that activate downstream signaling. Upstream of RAS, endogenous mitogens such as epidermal growth factor (EGF), that are enriched in the tumor microenvironment, bind to cell surface receptor tyrosine kinases (RTKs) and activate a cascade of events, starting with removal of GDP from WT RAS isozymes by guanine nucleotide exchange factors (GEFs). When in a nucleotide-free conformation, high intracellular concentrations of GTP rapidly bind and switch RAS “on”, to stimulate MAPK/AKT signaling, culminating in the transcription of genes that encode for proteins essential for normal cell turnover and replacement (6). RAS mutations result in appreciably slower rates of GDP/GTP exchange caused by preventing GTPase activating proteins (GAPs) from removing GTP to turn RAS “off”, resulting in hyperactivation of downstream MAPK/AKT signaling (6). WT RAS isozymes, NRAS and HRAS, are co-expressed in KRAS mutant cancer cells whereby their proliferation can be driven not only by mutant KRAS, but also by extracellular mitogens that activate WT RAS isozymes. Nonetheless, KRAS^G12C^ inhibitors have been shown to have exquisite selectively in inhibiting the growth of tumors harboring KRAS^G12C^ and, consequently, would not be expected to affect the growth of tumors with other KRAS mutations (or other RAS isozymes). However, unchecked activity from WT RAS isozymes might contribute to intrinsic or acquired resistance. Further, because KRAS^G12C^ mutations only account for 3% of CRC cases, there is an urgent medical need to treat CRC patients harboring other KRAS mutations, including patients with G12D (30.1%), G12V (24.2%), G12R (2.1%), or other (19.6%) mutations (7). Thus, a pan-KRAS inhibitor would be expected to have broader use for CRC and other RAS driven cancers given its additional potential to circumvent resistance from unchecked activity of WT RAS isozymes. Nonetheless, both approaches would require a mechanism to selectively inhibit mutant RAS in cancer cells without affecting the activity of WT RAS in normal cells, essential for turnover and replacement in rapidly dividing tissues.

## RAS signaling

As a membrane bound small guanine nucleotide binding protein, RAS can readily switch between an active GTP-bound state and an inactive GDP-bound conformation under normal physiological conditions. This cascade is modulated by RTKs whereby dimerization is induced by ligand binding. Receptor dimerization leads to the activation of intrinsic tyrosine kinase and autophosphorylation of tyrosine residues. The phosphorylated receptor interacts with GRB2 (growth factor receptor bound protein 2) and GEFs such as SOS (Son of Sevenless), that catalyze GDP/GTP exchange, leading to the active conformation of RAS. RAS-GTP activates several pathways such as RAF-MEK-ERK and PI3K-AKT-mTOR promoting cell proliferation and survival. In normal cells, RAS is switched off by GAPs inducing GTP hydrolysis and forming inactive RAS-GDP. But this is impeded in cancer cells by the inability of GAPs to bind RAS, thereby reducing the hydrolysis of GTP, favoring RAS to be in a constitutively activated conformation (8).

The KRAS protein has a molecular weight of 21 KD and is composed of six beta strands and 5 alpha helices, which form 2 major domains, referred to as the G-domain and the C-terminal (9). The G-domain, which is highly conserved contains the switch I and switch II loops that are responsible for GDP/GTP exchange (10).

**Figure 1:**
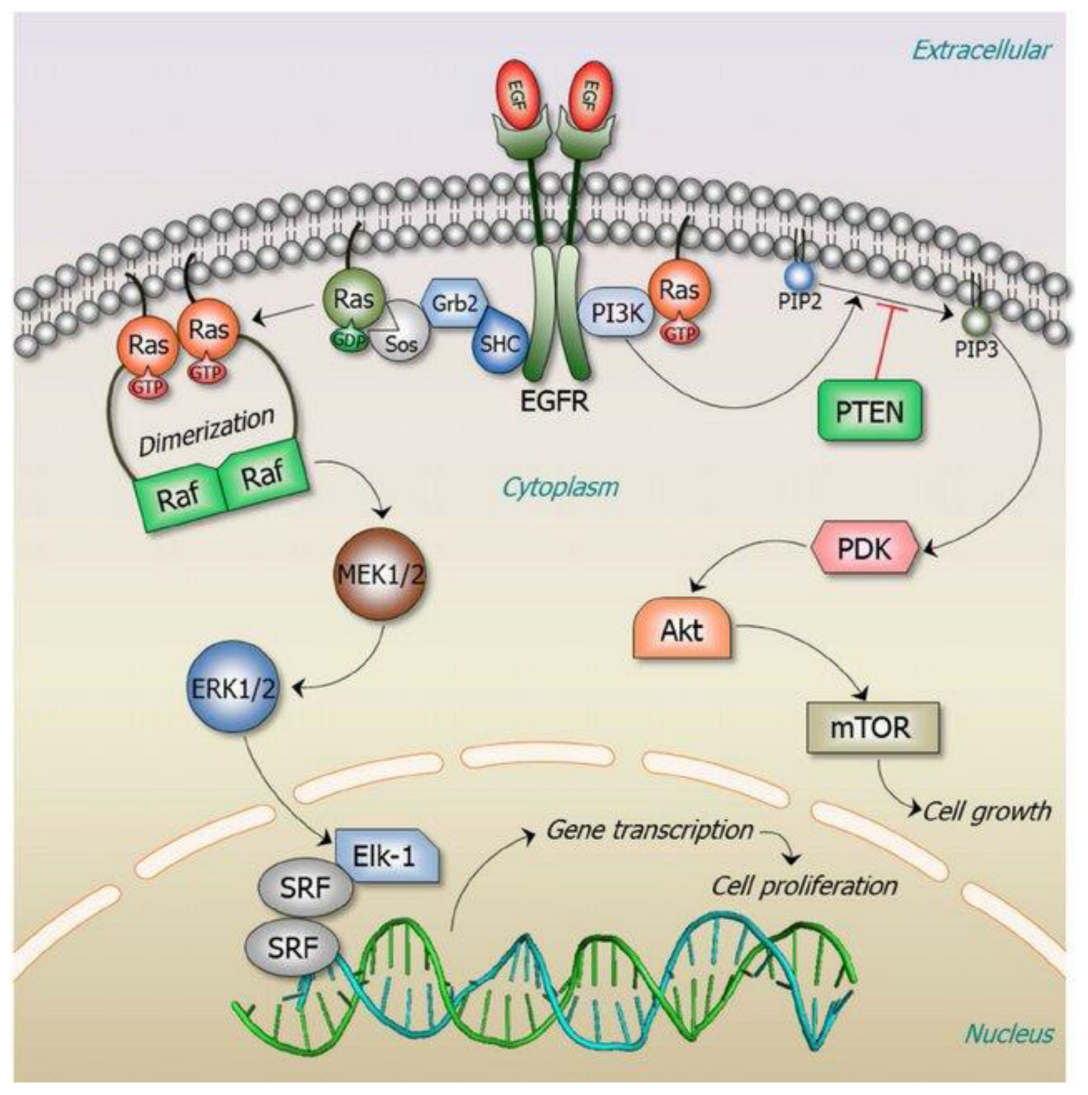
RAS signaling is versatile as it involves numerous cellular functions. The key RAS effector pathway is the mitogen-activated protein kinase (MAPK), Raf-MEK-ERK pathway (11). Reproduced here from Oncotarget (Ruth Nussinov et al., 2014) under Creative Commons Attribution license.

**Figure 2:**
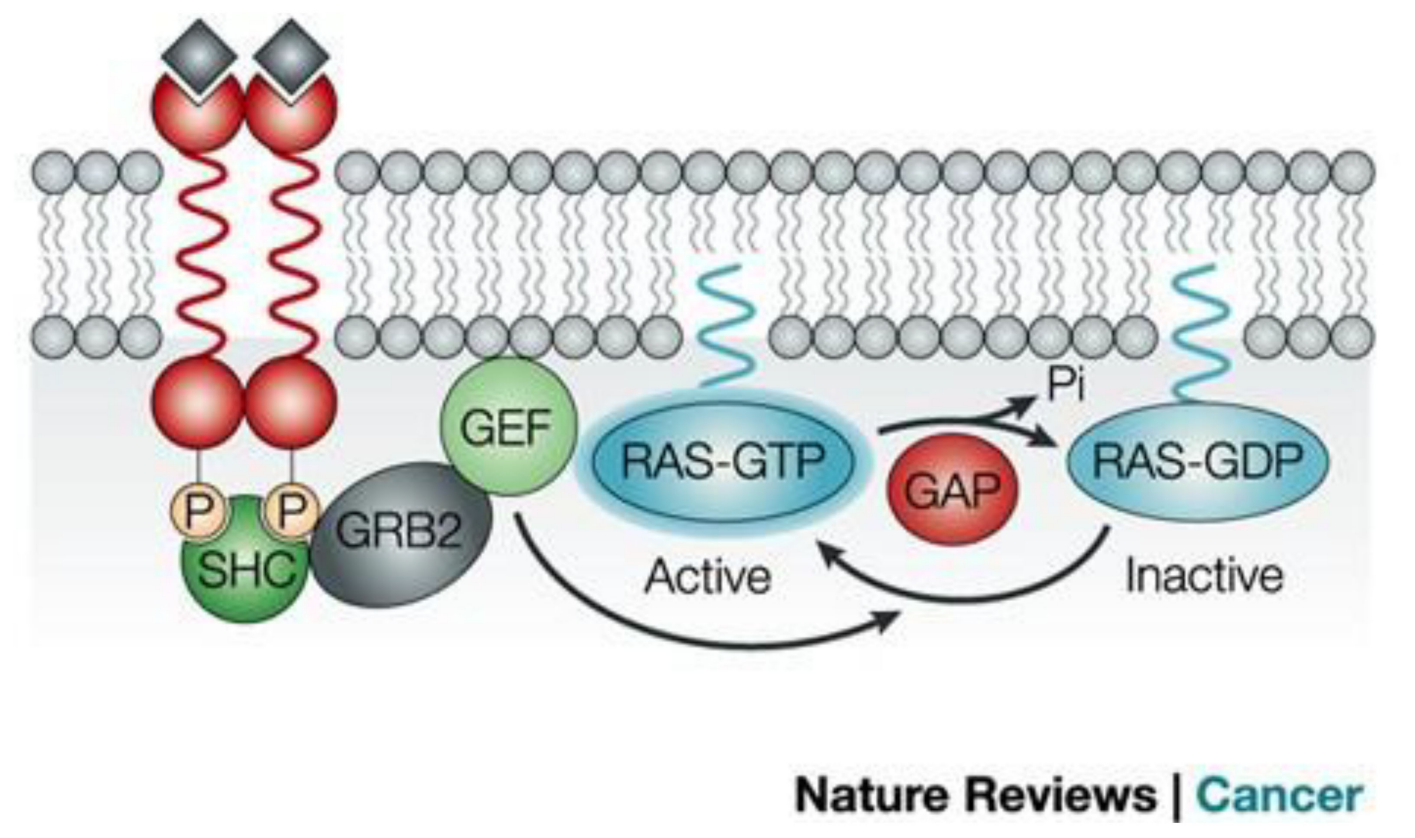
Signaling upstream of RAS (12). The RAS activation is controlled by the cycle of hydrolysis of bound GTP, catalyzed by GTPase activating proteins and the replacement of bound GDP with fresh GTP, which is catalyzed by guanine nucleotide exchange factors. Reproduced here from Nature Reviews Cancer (Julian Downward 2003) under Creative Commons Attribution license.

**Figure 3:**
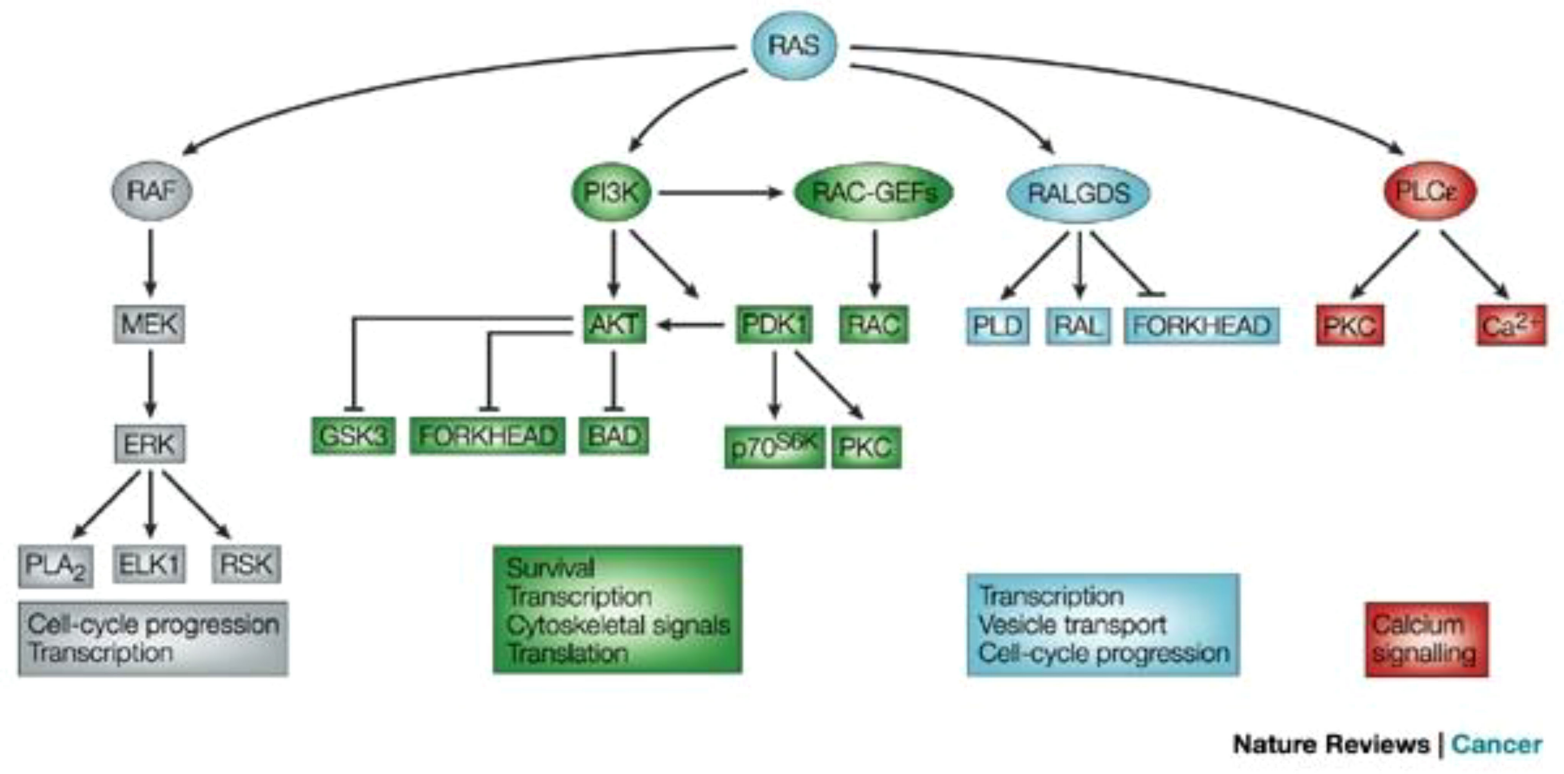
Signaling downstream of RAS (12). The main effector proteins with which RAS interacts, once in its active GTP-bound state, is shown. Reproduced here from Nature Reviews Cancer (Julian Downward 2003) under Creative Commons Attribution license.

**Figure 1** illustrates the RAS signaling pathways. The upstream and downstream signaling mechanism of RAS are depicted in **Figures 2**, **3** respectively.

## Development of KRAS^G12C^ inhibitors for colorectal cancer

Until recently, KRAS was considered undruggable as the protein apparently lacked deep pockets for small molecule binding, apart from the nucleotide binding domain (13). In addition, the high affinity of KRAS for GTP makes it difficult to develop competitive small molecule inhibitors to block GTP activation of RAS. Despite these challenges, multiple attempts have been made to discover small molecules to directly inhibit RAS. Early clinical trials of Sotorasib (the first FDA approved KRAS^G12C^ inhibitor) in CRC patients with a KRAS^G12C^ mutation resulted in lack of response as well as in non-small cell lung cancer (NSCLC) patients with the same mutation. For example, Sotorasib monotherapy in CRC KRAS^G12C^ patients previously treated with fluoropyrimidine, oxaliplatin, and irinotecan, demonstrated 9.7% objective response rate (ORR) in 62 patients (14). Another study evaluated the effect of Adagrasib (the second FDA approved KRAS^G12C^ inhibitor) in 43 CRC patients in KRYSTAL-1 (NCT03785249) trial (**Table 1**) which demonstrated 19% response rates (15). The reasons underlying the lower response to the drugs in CRC targeting G12C may be the rapid development of treatment related adaptive signaling resistance as these tumors undergo significant rebound in ERK phosphorylation (16). Another hypothesis for this contradictory response is the presence of higher levels of upstream receptor tyrosine phosphorylation compared to NSCLC, specifically in EGFR (16). Thus, combination therapies with small molecule KRAS inhibitors and anti-EGFR monoclonal antibodies have gained traction recently. In line with this, CodeBreak 101 (NCT04185883) reported an overall survival of 30% in 40 metastatic CRC patients (**Table 1**) treated with Panitumumab and Sotorasib combination therapy (17). Similarly, Adagrasib in combination with Cetuximab demonstrated an ORR of 46% compared to Adagrasib monotherapy with ORR of 19% (18).

**Table 1:** Summary of key clinical trials of KRAS^G12C^ inhibitors in CRC, advanced solid tumors, and NSCLC patients.

CLINICAL TRIAL	NUMBER OF PATIENTS	INTERVENTION	ORR (95% CI)	MEDIAN PFS (95% CI)
NCT03600883 CodeBreak100 Phase II	62	Sotorasib	9.7%	10.6 months
NCT04185883 CodeBreak 101 Phase I	40	Sotorasib + Panitumumab	30%	Not reported
NCT03785249 KRYSTAL-1 Phase I	43	Adagrasib	19%	5.6 months
NCT03785249 KRYSTAL-1 Phase I	28	Adagrasib + Cetuximab	46%	6.9 months
NCT04613596 KRYSTAL-7	53	Adagrasib + Pembrolizumab	49%	Not reported

## Mechanisms of resistance to KRAS^G12C^ inhibitors

The limited response of CRC patients to KRAS^G12C^ inhibitors may be attributed to multiple mechanisms of resistance, both upstream and downstream of KRAS as well as co-lateral pathways (e.g., Wnt/β-catenin) that can compensate for the effects of a mutant specific KRAS inhibitor. In addition, co-occurring mutations such as G13D, R68M, and A59S/T confer resistance selectively to Sotorasib, while Q99L alteration is selective to Adagrasib (19). The most common X96D/S mutation confers strongest resistance to both drugs (20). A frequently identified mechanism of resistance that diminishes the therapeutic efficacy of KRAS inhibitors is the induction of bypass MAPK signaling to overcome KRAS blockade. Initial studies revealed significant suppression of negative regulators of MAPK signaling and that ERK dependent signaling is reactivated to bypass KRAS^G12C^ treatment. Further insights into resistance mechanism suggest that only the cells with KRAS^G12C^ in the inactive confirmation are strongly inhibited by novel KRAS^G12C^ inhibitors. This leads to non-uniform rates of inactive to active KRAS^G12C^ cycling. Subsequently, these cells with KRAS^G12C^ preferentially held in active confirmation could be insensitive to treatment and could mediate reactivation of MAPK signaling (21, 22).

Furthermore, CRC cells show early development of adaptive resistance to KRAS^G12C^ inhibitors by rapid upregulation of p-MEK and p-ERK and increased basal phosphorylation and activation of EGFR. CRC cells respond to EGF stimulation by activating RAS-MAPK signaling even in the presence of an activating KRAS^G12C^ mutation, which contrasts with NSCLC cells. In line with this, preclinical studies suggest that primary resistance to KRAS inhibition is less likely, and the predominant issue appears to be drug-induced (acquired) resistance. This contrasts with NSCLC where the key issue is primary (intrinsic) resistance. In summary, EGFR specifically mediates adaptive resistance response in CRC cells. Finally, resistance is also observed by induction of epithelial to mesenchymal transition in conjunction with increased PI3K/AKT signaling due to upregulated EGFR signaling and subsequently leading to increased MAPK signaling *via* FGFR (23).

Thus, increased RTK signaling coupled with other mechanisms such as increased GTP-bound KRAS^G12C^ leads to tumor progression and triggers further downstream signaling. Alternate pathways such as Wnt/β-catenin signaling is activated interacting with mutant KRAS signaling further promoting oncogenic signaling and increased resistance (24).

Primary resistance also plays a role in lack of efficacy of KRAS inhibitors driven by multiple mechanisms. This includes rapid adaptive feedback RTK-RAS-MAPK reactivation signaling upon deficit host immune system. The formation of active GTP-bound KRAS^G12C^ from non-uniform cycling between GTP-bound active and GDP-bound inactive states driven by EGF and persistent upstream RTK activity with signaling through alternative wild-type RAS forms in CRC. The induction of EMT and disinhibition of cell-cycle transition by co-occurring alterations in CDKN2A also contribute to low efficacy of KRAS inhibitors. The differences in pharmacokinetic properties of different KRAS inhibitors also contribute to low efficacy of some of these inhibitors (25).

## Early phase clinical trials of KRAS^G12C^ inhibitors

The first human phase 1 trial on Sotorasib at a daily dose of 960 mg in 42 CRC patients (CodeBreak 100, NCT03600883) demonstrated modest clinical activity (ORR of 7.1%) compared to NSCLC patients with ORR of 32.3% (26). The phase 2 CodeBreak 100 trial with the same dose of Sotorasib demonstrated an ORR of 9.7% in patients with metastatic KRAS^G12C^ mutant CRC with prior fluoropyrimidine, oxaliplatin, and irinotecan treatment (14). The KRYSTAL-1 phase 1/2 trial (NCT03785249) investigating Adagrasib in patients previously treated with chemotherapy or anti-PD1 showed ORR of 22% and disease control rate (DCR) of 87% in 45 CRC patients (15).

## Current clinical trials involving KRAS^G12C^ inhibitors

There are 12 different KRAS^G12C^ inhibitors currently under clinical investigation. In total, there are 76 entries for KRAS^G12C^ trials, out of which 39 were trials in CRC patients, while 33 were trials involving lung cancer patients. (Two trials were excluded as they are not specific to KRAS^G12C^ mutant tumors, and 2 others were excluded as they are only diagnostic studies).

Noteworthy, Novartis JDQ443 binds KRAS without involving H95 residue and maintains activity among tumors with a dual G12C and H95 KRAS mutation. This non-selectivity may reduce or alleviate acquired resistance (27, 28). On a similar note, JNJ-74699157 binds near the switch II pocket through a different cysteine residue interaction and may also mitigate resistance (29). More recently, a striking finding on Eli Lilly’s LY3537982, demonstrated that this novel KRAS inhibitor in combination with Cetuximab showed 45% ORR in 11 CRC patients (30). As would be expected, combinations of KRAS^G12C^ inhibitors and inhibitors of other downstream components of RAS/MAPK pathways such as BRAF or MEK offer promising approaches as well (31). Another combination strategy was based on KRAS inhibition triggering pro-inflammatory changes in the tumor microenvironment. This was shown by combining anti-PD1 therapy and Sotorasib, which demonstrated increased CD8+ T-cell infiltration in the tumor microenvironment and promising efficacy (32).

**Figure 4:**
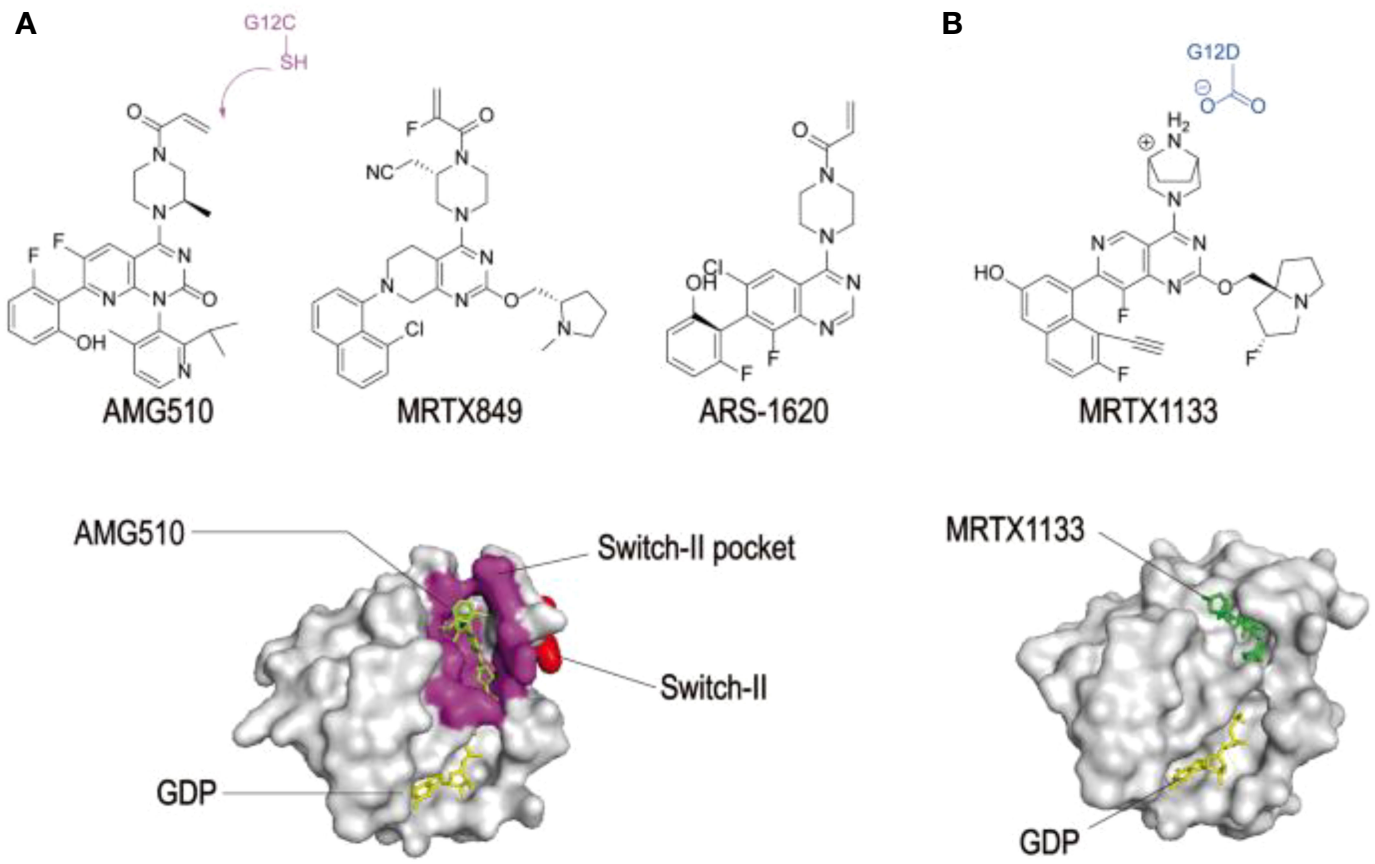
Structures of KRAS surfaces targeted by KRAS mutant inhibitors. a Switch-II pocket (purple) of KRAS (G12C) bound to AMG510 (PDB: 6OIM). b MRTX1133 with KRAS G12D/GDP (PDB: 7RPZ). (33) Reproduced here from Molecular Cancer (Weidong Zhang et al., 2022) under Creative Commons Attribution license.

**Figure 4** shows the structure of KRAS surfaces targeted by KRAS inhibitors. a) switch II pocket of KRASG12c bound to AMG510 b) MRTX1133 with KRASG12D/GDP.

## Combination treatment strategies

Considering mechanisms of resistance upstream of RAS, combined treatment of Sotorasib or Adagrasib with EGFR inhibitors is currently being evaluated in clinical trials. Preclinical studies reported that the EGRF inhibitor, Cetuximab, sensitizes KRAS^G12C^ mutated CRC cell lines to Sotorasib, leading to sustained downregulation of phosphorylated MEK and ERK proteins, causing cell proliferation arrest and apoptosis (16). The KRYSTAL-1 (NCT03785249) trial conducted in 28 CRC patients reported ORR of 46% and DCR of 100% in patients treated with Cetuximab and Adagrasib (16).

CodeBreak 101 umbrella trial tested Sotorasib with inhibitors of MEK, CDK4/6, mTOR, or VEGFR in additional cohorts. Similarly, KRYSTAL-1 trial is also exploring similar combination strategies. Combinations of Sotorasib and the MEK inhibitor, Trametinib were tested in 18 CRC patients with promising efficacy and safety (34). Similarly, KRAS^G12C^ inhibitors in combination with CDK4/6 inhibitors such as Palbociclib demonstrated significant downregulation of KRAS pathway phosphorylation (35). Another emerging strategy includes simultaneously targeting other components of the KRAS pathway. One such target is the SHP2 which promotes KRAS signaling and CRC progression. SHP2 inhibition increases GDP-bound KRAS^G12C^ and shows synergism with KRAS inhibitors *in vitro* (36, 37).

Ongoing phase 1 trials are actively progressing on novel SHP2 inhibitors such as TN0155, BBP-398 and RMC-4630 with plans to test in combination with KRAS inhibitors (38–40). BI-3406, an SOS1 inhibitor demonstrates increased response in combination with Trametinib (20).

A phase 1b trial (NCT04449874) reported the activity of Divarasib, a covalent KRAS^G12C^ inhibitor (**Table 2**) that turns off oncogenic signaling by irreversibly locking the protein in an inactive state, and Cetuximab in 29 CRC patients with KRAS^G12C^ mutation. The ORR was 62.5% and median duration of response was 6.9 months. This encouraging anti-tumor activity of the combination therapy supports further investigation (41). Strikingly, Divarasib is also shown to be 5 to 20 times more potent and fifty times more selective than Sotorasib and Adagrasib (42).

**Table 2:** A summary of registered trials listed on clinicaltrials.gov. as of March 24, 2024.

Clinical candidate	Clinical trial	Sponsor
BPI-421286	NCT05315180	Betta Pharmaceuticals
D3S-001	NCT05410145	D3 Bio (Wuxi) Co.
Jab-21822	NCT05002270	Jacobio Pharmaceuticals
JNJ-74699157	NCT04006301	Janssen
GFH925	NCT05756153	Zhejiang Genfleet Therapeuticals
HBI-2438	NCT05485974	Huyabio International
JDQ443	NCT05445843	Novartis Pharmaceuticals
YL-15293	NCT05119933	Shanghai Yingli Pharmaceutical
GDC-6036	NCT04449874	Genentech
MK-1084	NCT05067283	Merck Sharp & Dohme
LY3537982	NCT04956640	Eli Lilly and Company
HS-10370	NCT05367778	Jiangsu Hansoh Pharmaceutical

KRYSTAL-10 (NCT04793958) is a randomized phase 3 trial to test Adagrasib (600mg BID) and Cetuximab (500mg) in patients with KRAS^G12C^ metastatic CRC. This combination therapy is being compared with standard chemotherapy receiving FOLFIRI (leucovorin calcium (folinic acid), fluorouracil, and irinotecan hydrochloride). Another phase 3 randomized trial NCT05198934 is testing Sotorasib and Panitumumab and comparing with Tipiracil or Regorafenib is underway (**Table 3**) in previously treated metastatic KRAS^G12C^ mutant CRC patients (43).

**Table 3:** A summary of current clinical trials in patients with KRAS^G12C^ mutant CRC listed on clinicaltrials.gov. as of March 24, 2024.

Trial	Treatment Arms
CodeBreak 300 (NCT05198934) phase III	Sotorasib + Panitumumab versus Investigator’s choice
KRYSTAL-10 (NCT04793958) phase III	Adagrasib + Cetuximab versus Folfox/Folfiri

## Other targets

BAY-293, an SOS1 inhibitor exhibits synergistic activity when combined with ARS-1620 in KRAS^G12C^ mutant CRC cancer cell lines proving that targeting the inactive GDP-bound form is a promising approach for generating anti-RAS therapeutics. Another novel SOS1 inhibitor BI-1701963 is currently under investigation as a single agent or in combination with Trametinib (NCT04111458) or Adagrasib (NCT04975256) (44, 45). Recently, BI-3406, another SOS1 inhibitor was demonstrated to be more potent and selective for inhibiting SOS1, decreasing KRAS-GTP levels and suppressing cancer cell proliferation (46). The tyrosine phosphorylation of SHP2 recruits GRB2-SOS complex promoting RAS nucleotide exchange acting as a scaffold protein. Currently, SHP2 inhibitors have gained attention and several of them are in the early phase of clinical trials. For example, RMC-4630 in combination with ERK inhibitors LY3214996 is in phase 1 clinical trial for KRAS^G12C^ CRC. (NCT04916236). Another inhibitor, TN0155 is in phase 1b/2 clinical trial (NCT0469918) in combination with KRAS^G12C^ inhibitor, JDQ443 in KRAS^G12C^ mutant CRC patients.

A phase 1 trial (NCT01085331) evaluated the effects of MEK inhibitor Pimasertib combined with FOLFIRI as a second line treatment of KRAS metastatic CRC. However, GI and skin toxicity were reported with Pimasertib (47). A novel RAF dimer inhibitor, Lifirefenib, demonstrated acceptable safety in phase 1 trials, but no activity was observed in KRAS mutant CRC patients (48). Three enzymes engage in post-translational modifications of KRAS which is the 1^st^ step of membrane localization, FTase, RCE1 and (Isoprenylcysteine carboxymethyltransferase) ICMT. Inhibitors of ICMT such as cysmethynil and UCM-1336 showed potential to inhibit KRAS activity and impair the growth of KRAS mutant cell lines (49). However, clinical data are not yet available in patients with KRAS^G12C^ mutant CRC.

### Current CRC treatment and limitations

The primary therapeutic strategy for resectable colorectal cancer is surgical removal and in non-resectable CRC, the strategies include chemotherapy, radiotherapy, and immunotherapy along with combination therapies. However, these approaches do not come without limitations such as relapse of acquired multi-drug resistance CRC. Recently, immune checkpoint inhibitors, T cell receptor alterations, cytokine therapy, RNA-based therapies such as siRNA and miRNA have yielded promising results (50).

### Radiotherapy

Two of the adjuvant radiotherapies, a short course and long course are currently available, which are better options for treating stage II and stage III CRC. However, acute toxicity rates are high with long course radiotherapy. Decreased toxicity is observed with new delivery methods such as intensity-modulated radiotherapy (51, 52).

### Chemotherapy

The commonly approved chemotherapy medications for stage III and IV CRC include fluoropyrimidines (capecitabine, Fluorouracil), topoisomerase I inhibitors (irinotecan, oxaliplatin) and tri-fluridine/tipiracil). After surgery for CRC, adjuvant fluoropyrimidine based chemotherapy is standard to reduce tumor recurrence and increases survival rate (53). Topoisomerase I inhibitor irinotecan and oxaliplatin are added to 5-flurouracil and folinic acid (leucovorin) as combination therapy regimens for metastatic CRC (known as FLOFOX and FOLFIRI) or to capecitabine (CAPOX). Regorafenib is an FDA approved tyrokinase inhibitor targeting VEGF, platelet derived growth factor, fibroblast growth factor in metastatic CRC (54).

### Target specific treatment

Monoclonal antibodies such as Cetuximab, Panitumumab etc. are epidermal growth factor receptor inhibitors, while Bevacizumab and Ramucirumab target vascular epidermal growth factor and its receptor respectively (55). Cetuximab and Panitumumab are FDA approved first line treatment for CRC (56). CTLA-4 inhibition could inhibit tumor progression by upregulating effector T cell activity and suppressing regulatory T cells. FDA approved low dose Ipilimumab in combination with Nivolumab is used for previously treated microsatellite instability-high and deficient mismatch repair metastatic CRC. Pembrolizumab and Nivolumab (PD1 inhibitors) are also used in CRC (57).

### Vaccines

Several clinical trials are conducted on introducing vaccines against CRC. The tumor associated antigens that are targeted include surviving, EGFR, VEGFR1 etc. These vaccines could activate local immune cells, releasing tumor antigens, increasing T cells and dendritic cell infiltration to the site of action (58).

Thus, in summary every patient has a unique tumor microenvironment, and individualized approaches to treating CRC are needed. Although conventional cytotoxic drugs are the first line of agents for CRC, their shortcomings include, toxicity and drug resistance leading to recurrent CRC. In addition to these, chemotherapy is associated with systemic toxicity, fever, stomatitis, mucositis, leukopenia, and thrombocytopenia. New approaches are emerging for treating CRC to overcome these drawbacks.

### RAS PROTAC study

Despite the clinical success of KRAS^G12C^ inhibitors, acquired resistance is the major drawback with these agents. RAS was considered undruggable initially due to its insufficient binding pockets. The 1^st^ half of the RAS protein is referred to as effector lobe (residues 1–85) while the second half of the G-domain (residues 86–166) is referred to as allosteric lobe. The exploration of high affinity macromolecular binders against the effector lobe potentially to inhibit RAS signaling is in the spotlight recently. Effective targets on the effector lobe include switch regions for which GDP and GTP specific binders have been identified (59). By genetically fusing E3-ligase subunits such as Von Hippel-Lindau tumor suppressor to monobodies NS1 and 12VC1, RAS degrader constructs were generated. These degraders have potent RAS signaling suppression and anti-proliferative activities (60).

These degraders emulate PROTAC (proteolysis targeting chimera) mode of action. Compared with competitive inhibitors, PROTACs instruct the degradation of protein by recruiting ubiquitin-proteasome system to target protein. They can therefore bind outside of an active protein site and after degradation abrogate any scaffolding functions of the target. This is attributed to their hybrid structure, containing one bonder (the warhead) for the target protein that is tethered via a linker to a moiety recuring the E3 ligase (61). PROTACs can be reused after reversible binding and degradation of target proteins. Current RAS targeting protacs (XX-4–88, LC-2, KP-14) are all built on covalent G12C inhibitor and cannot be beneficial as these inhibitors are consumed due to covalent cysteine engagement (62).

A remarkable development in this line is the reversible covalent inhibitor YF135 which employs cyanoacrylamide for cystine linkage (63). However, optimization of linker length requires further research and developmental efforts (64).

Given the spatial temporal distinct expression of E3 ligase in tissues and cells. PROTACs may provide a more controlled drug action and it remains to be seen if any of the RAS ligands can be converted to PROTACs.

### Japanese guidelines for KRAS^G12C^

The increase in targeted therapy for CRC based on genomic status has led to the clinical development of new agents which could be potentially used in patients with microsatellite instability and/or mismatch repair and metastatic CRC (mCRC) due to BRAF^V600E^ mutations. In Japan, Trastuzumab combined with Pertuzumab was approved in March 2022, for ERBB2 (ErbB2 Receptor Tyrosine Kinase 2) positive mCRC. This development devised a better strategy for precision oncology for rare genomic alterations. The tumor genomic status in mCRC was determined for KRAS and NRAS, BRAF^V600E^ mutations, ERBB2 and microsatellite instability (MSI)/mismatch repair (MMR) (65, 66).

The SCRUM-Japan GI-SCREEN was launched in Japan by the National cancer center hospital East in 2015. Approximately, 30,000 patients were screened using tissue and plasma assays in this nationwide screening project. In 2017, the regulatory graded registry platform (SCRUM-Japan-Registry) was established to collect efficacy data of standard therapy in patients with rare molecular alterations. Their treatment strategy was based on the 4 genomic status, along with the primary tumor location (67, 68).

In this SCRUM-Japan-GI-screen, the phase II TRIUMPH study, demonstrated the efficacy of Trastuzumab plus Pertuzumab in patients with ERBB2 amplification and this study showed ORR of 30% in 27 patients who were ERBB2 positive in tissues (69). Another interesting ongoing randomized multicenter phase II trial SWOG S1613 is recruiting patients with RAS and RAF wild type ERBB2 positive mCRC who received at least one prior line of therapy. The aim of this study is to compare the efficacy of trastuzumab plus Pertuzumab versus Cetuximab plus Irinotecan (NCT03365882) (65). A combination of chemotherapy plus anti-VEGF therapy along with immune oncology therapy could potentially be more effective in triggering immunogenic cell death and release of tumor antigens (70). In summary, for patients with MSI/MMR or BRAF V600E mCRC, Pembrolizumab is the first line therapy and Encoratinib plus Cetuximab with or without Binimetinib is considered as the second line therapy. New agents are proposed for rare molecular fractions such as ERBB2 amplification. While the efficacies of Trastuzumab plus Pertuzumab were indicated in single -arm trails, no anti-ERBB2 therapies are approved in the United States and European Union (65).

## Conclusion

The understanding of KRAS signaling, structural biology, and biochemistry over the last several years has led to FDA approval of the first direct acting KRAS^G12C^ inhibitors. While KRAS^G12C^ inhibitors are efficacious for NSCLC, their use for CRC faces challenges due primarily to the development of resistance. Although the monotherapy response rates remain low in CRC patients, combination therapies are more promising. Another option recently explored are the pan-KRAS and pan-RAS inhibitors which inhibit RAS regardless of the mutated allele, or in the latter case, also independent of the RAS isozyme, which may compensate for effects of mutant specific KRAS inhibitors (71, 72).

The development of KRAS^G12C^ inhibitors for CRC is ongoing and larger randomized clinical trials may reveal more promising approaches. The recent development of KRAS^G12C^ targeted therapy in CRC has clearly ignited the field to develop new RAS inhibitors potentially with broader scope and reduced potential for resistance.

## Author contributions

GP: Conceptualization, Investigation, Writing – original draft, Writing – review & editing. PC: Conceptualization, Investigation, Writing – original draft, Writing – review & editing. YM: Investigation, Validation, Writing – review & editing. KB: Investigation, Validation, Writing – review & editing.
